# New Trends in Cancer Therapy: Targeting Ion Channels and Transporters

**DOI:** 10.3390/ph3041202

**Published:** 2010-04-20

**Authors:** Annarosa Arcangeli, Andrea Becchetti

**Affiliations:** 1 Department of Experimental Pathology and Oncology, University of Florence, Italy; 2 Department of Biotechnology and Biosciences, University of Milano-Bicocca, Italy

**Keywords:** oncology, hERG, K_v_11, K_v_10, glioma, leukemia, Na^+^/K^+^ ATPase, side effects

## Abstract

The expression and activity of different channel types mark and regulate specific stages of cancer establishment and progression. Blocking channel activity impairs the growth of some tumors, both *in vitro* and *in vivo*, which opens a new field for pharmaceutical research. However, ion channel blockers may produce serious side effects, such as cardiac arrhythmias. For instance, K_v_11.1 (hERG1) channels are aberrantly expressed in several human cancers, in which they control different aspects of the neoplastic cell behaviour. hERG1 blockers tend to inhibit cancer growth. However they also retard the cardiac repolarization, thus lengthening the electrocardiographic QT interval, which can lead to life-threatening ventricular arrhythmias. Several possibilities exist to produce less harmful compounds, such as developing specific drugs that bind hERG1 channels in the open state or disassemble the ion channel/integrin complex which appears to be crucial in certain stages of neoplastic progression. The potential approaches to improve the efficacy and safety of ion channel targeting in oncology include: (1) targeting specific conformational channel states; (2) finding ever more specific inhibitors, including peptide toxins, for channel subtypes mainly expressed in well-identified tumors; (3) using specific ligands to convey traceable or cytotoxic compounds; (4) developing channel blocking antibodies; (5) designing new molecular tools to decrease channel expression in selected cancer types. Similar concepts apply to ion transporters such as the Na^+^/K^+^ pump and the Na^+^/H^+^ exchanger. Pharmacological targeting of these transporters is also currently being considered in anti-neoplastic therapy.

## 1. Introduction

Evidence tracing back to the seventies indicates that ion channel blockers, especially for K^+^ and Cl^−^channels, impair neoplastic cell proliferation. These observations were subsequently extended, thus opening a wide field of research on the functional implications of ion channels in the biology of cancer cells [1,2]. Prominent alterations in the expression and activity of ion channels and transporters occur in neoplastic cells. These changes, besides contributing to the promotion of tumorigenesis overall, may mark and stimulate specific progression stages [3]. In agreement with the many observations carried out *in vitro*, recent studies indicate that blocking the activity of certain voltage-gated channels impairs the growth of some tumours *in vivo* [4,5,6,7]. Encouraging results have also been obtained with inhibitors of the Na^+^/K^+^ pump in glioblastoma cells grafted in mice [8]. Analogous approaches are currently under test in rodent models that may have relevance for cancerogenesis, such as chronic inflammatory autoimmune diseases [9].

These studies open new vistas for pharmaceutical research. In principle, targeting ion channels presents considerable advantages for cancer treatment, such as the possibility of convenient (and potentially less toxic) extracellular access. However, ion channels normally exert disparate physiological roles in excitable as well as non-excitable cells. Therefore, channel targeting may produce serious side effects. A clear example, fully discussed below, is provided by K_v_11.1 (hERG1) channels. These normally contribute to the repolarization phase of the cardiac action potential and blocking them may lead to fatal arrhythmias, usually caused by lengthening of the QT interval. However, K_v_11.1 is also frequently expressed in cancer cells [10] and treatment with hERG1 blockers *in vivo* appears to have therapeutic effects for leukemias [6,7]. It is thus clear that proper calibration of therapy or, even better, production of compounds that preferentially target the channels expressed by tumour cells are experimental tasks that merit further study.

Recently, we have extensively reviewed the manifold aspects of ion channel expression and function in different tumours [3]. In the following paragraph, we provide a brief update of the field. A rough synopsis of ion channels and transporters involved in oncology is shown in Table 1. Next, we focus on the main pharmacological issues, by devoting special attention to the possible strategies for targeting ion channels in cancer and circumventing the side effects.

## 2. Channel Expression in Tumour Cells: An Update

K^+^ channels have attracted most of the work in the field since the early discovery that they often control the proliferation of non-excitable cells. These observations were accompanied by many studies on the expression and function of K^+^ channels in different tumours, and particularly hematologic malignancies. Further investigations have aimed at understanding the contribution of specific channel types to the neoplastic progression. The control of cancer cell proliferation in different experimental models is often modulated by voltage gated K^+^ channels (VGKCs; in particular K_v_1.3, K_v_10.1 and K_v_11.1), Ca^2+^-dependent K^+^ (K_Ca_) channels (especially K_Ca_1.1 and K_Ca_3.1) and two-pore (K_2p_) channels (in particular K_2p_2.1). At least in the case of VGKCs and K_Ca_, such control can occur through the modulation of membrane potential (V_m_), which in turn regulates transmembrane Ca^2+^ flow. This mechanism has been proven in breast, prostate and colon cancers and in melanoma, and it may have general importance [1,2,3]. The phenotype of different cell types is characterized by a specific “calcium signature” dependent on the kinetics, the magnitude and the sub-cellular localization of calcium signals. In turn, intracellular Ca^2+^ levels participate to the control of cell cycle checkpoints, in normal and neoplastic proliferation [11,12,13]. It has been repeatedly observed that a dysregulation of the calcium signature in cancer can be mainly attributed to Transient Receptor Potential channels (TRP channels), whose role in tumor progression is increasingly recognized [14,15]. In prostate cancer, for example, classic (TRPC), vanilloid (TRPV) and melastatin (TRPM) TRP channels are all involved, through different mechanisms, in the development of androgen independence. This leads to apoptosis resistance and hence treatment failure [16]. Besides the roles in volume control and cell motility that will be described below, TRP channels’ expression could also represent a useful prognostic marker, at least in prostate cancer [17]. Finally, TRP channels are involved in vascular permeability and angiogenesis, with clear implications for tumour growth and metastasis formation [18,19]. 

Another mechanism by which K^+^ channels control tumor progression is the regulation of cell volume, which requires interplay with Ca^2+^ and Cl^−^ channels. At least three types of swelling-activated channels are implicated in cancer cell physiology: the volume-regulated anion channels (VRAC), the swelling-activated K^+^ channels TASK-2 and K_Ca_1.1, and the volume sensitive TRP Ca^2+^ channels, such as TRPV4 and TRPM4 [20]. Cell volume control plays a central role in three crucial aspects of cancer cell biology: cell proliferation, apoptosis and migration. Activation of volume-sensitive K^+^ and Cl^−^ channels underlies the volume decrease which is an early and necessary part of the apoptotic response. Moreover, pioneering work carried out in H. Sontheimer’s laboratory determined the role of K_Ca_1.1 (hsloBK) channels, which operate in a finely coordinated manner with Cl^−^channels to regulate glioma cell invasiveness and metastasis [21]. In particular, ClC-3 is crucially involved in the invasive process [22]. This channel type is inhibited indirectly, but specifically, by a scorpion toxin, chlorotoxin (Cltx), which suggested a pharmacological approach that exploits a radiolabelled Cltx compound (I131-Cltx; [23]), as is illustrated in more detail later. The important contribution of K_Ca_3.1 channels to the regulation of cell migration has also been pointed out. To accomplish this function, K_Ca_3.1 operates in concert with other channels and transporters. The current model envisions ion transport proteins as supporting migration by inducing localized cell volume changes, swelling at the front and shrinkage at the rear. Volume changes at the rear end were proposed to be mediated by K_Ca_3.1 and volume-regulated anion channels (VRAC) [24,25]. In addition, transport proteins like the Na^+^/H^+^ exchanger NHE1 generate a characteristic pH nanomilieu at the cell surface that promotes the formation and release of cell-matrix contacts at the front and rear part of the cell, respectively [26,27]. Ion transporters like NHE1 are also required for persistent directional migration. Finally, TRPC1 channels play an important role in directed cell migration by regulating Ca^2+^ signaling events within the lamellipodium (reviewed in [24]).

K_v_11.1 also is implicated in motility and cancer invasion [28,29,30], besides the contribution it gives to regulate proliferation in leukemia [31,32], neuroblastoma [32] and melanoma cells [30]. The mechanism, however, appears to be different from that operated by K_Ca_3.1. K_v_11.1 coassembles with the β_1_ subunit of integrin receptors, thus forming a macromolecular complex that modulates downstream signalling pathways, such as tyrosine kinases and GTPases [33]. In this way, K_v_11.1 controls several aspects of cell physiology, such as migration and survival.

VGKCs often affect also the very first steps of tumor development, as is proven by the frequent up-regulation of VGKC transcripts following treatment with chemical carcinogens [34]. Hence, the expression of VGKCs and other structurally related K^+^ channels can be exploited for prognostic purposes in human cancers. For instance, Ousingsawat *et al.* [34] reported that VGKCs are associated with a poor prognosis in colorectal cancer, whereas K_IR_4.1, K_IR_3.1 and K_2p_2.1 correlate with tumor grade in gliomas, breast and prostate cancers, respectively [3].

The expression of voltage-gated sodium channels (VGSCs) has also been observed to increase in many cancer types, including breast, prostate, lung (both small-cell, SCLC, and non-small-cell, NSCLC), cervical cancer, leukaemia (reviewed in [3]) and mesothelioma cells [35]. Neoplastic cells mainly express the embryonic forms of VGSC (Na_v_ 1.5 in breast and Na_v_1.7 in prostate). In general, VGSCs stimulate some of the cell processes necessary for the metastatic cascade to proceed, although the precise mechanisms are still unclear [36]. In breast, prostate, and NSCLC tumor cells, VGSC activity increases invasiveness by stimulating cysteine cathepsin activity [37]. Non-conductive roles of VGSCs, with possible oncological relevance, such as direct involvement in cell adhesion, are also emerging [38]. As is the case of K^+^ channels, determining VGSC expression (particularly of Na_v_1.7) is also useful for prognostic purposes, particularly in prostate cancer [39].

Recent evidence shows that ligand-gated channels are also implicated in neoplastic progression. In particular, the nicotinic acetylcholine receptors (nAChRs) are ionotropic receptors typically activated by acetylcholine and nicotine at concentrations between 100 nM and 1 mM. NAChRs regulate cell proliferation, apoptosis and angiogenesis in several tumours, including lung cancers [40,41,42,43,44]. This is suggestive because smoke is an established risk factor for cancer, and particularly lung cancer. Activation of nAChRs stimulates (directly and indirectly) Ca^2+^ influx, which triggers the release of growth factors and other transmitter molecules. These produce autocrine and paracrine effects that promote proliferation, inhibit apoptosis and stimulate angiogenesis. Moreover, nicotine confers resistance to the chemotherapeutic-induced apoptosis. These effects occur in both SCLC and NSCLC cells, although the intracellular signalling cascades and the nAChR types involved are different. Hence, the molecular network centered on nAChRs is currently considered a promising target for the tobacco-related cancer therapy [45]. Interestingly, from the present perspective, radioligand competition data suggest that several carcinogens produced by tobacco inhalation, namely 4-(methylnitrosamino)-1-(3-pyridyl)-1-butanone (NNK), *N*-nitrosonornicotine (NNN) and diethylnitrosamine (DEN), bind with high affinity to nAChRs [46]. It has thus been proposed that some of the oncogenic effects of these compounds depend on specific activation of nAChRs, which may supplement the well-known effects caused by DNA targeting of several nitrosamine metabolites. Most data concern NSCLC cells, in which nAChRs not only exhibit an altered expression [42], but have been shown to stimulate cell proliferation through up-regulation of signalling pathways downstream to integrins [47]. 

Finally aquaporins (AQPs) have been recently studied in relation to cancer. AQPs are water channel proteins that facilitate transmembrane water flux. Ectopic AQP expression seems associated with several human cancers [48,49,50]. Molecular and biochemical studies have begun to clarify the role of AQPs in carcinogenesis. For example, AQP1 is implicated in both angiogenesis and cell cycle control [51,52,53], whereas AQP5 induces many phenotypic changes characteristic of cell transformation in fibroblasts in which it is ectopically expressed [54,55]. The effects of AQPs appear to be mediated by the signalling pathways that include Ras, which is induced by phosphorylation of the PKA consensus site of AQP5. In colon cancer cells, for instance, AQP5 regulates Ras, ERK and RB [55]. Therefore, the general intracellular mechanisms seem similar to those frequently associated to the function of ion channels. Finally, AQP5 promotes epithelial-mesenchymal transition in bronchial epithelial cells, and, consistently, shows a significant association with disease progression in NSCLC [50].

Ion pumps constitute other active players in the neoplastic cell physiology, potentially useful for therapeutic targeting. Thorough work has been carried out by R. Kiss’s group on the Na^+^/K^+ ^ATPase, which turns out to be a potentially useful target for oncologic therapy. The pump’s α1 subunit is overexpressed in some cancers [56], such as NSCLC [57] and glioblastomas [58,59]. In the latter, the Na^+^ pump is directly involved in the control of cell migration, thus cooperating with Cl^−^channels and aquaporins [60]. What is relevant, from the clinical point of view, is that inhibiting migration of highly migrating/invading glioma cells significantly increases their sensitivity to pro-apoptotic drugs. In other words, a potential way to overcome apoptosis resistance is decreasing glioma cell invasiveness [59,60,61]. Current therapeutic approaches based on these notions are discussed later.

### Ion transporters and the control of pH

Since Warburg’s classic work [62], potent control of intra- and extracellular pH has been known to be a major feature of cancer cells, so much so that it can be considered as one of the cancer hallmarks [63]. Since the late eighties, work from Serrano and co-workers in fibroblasts showed that proton pumps confer increased proliferation rate and resistance to acidic environment. Maintenance of a relatively alkaline cytosol, through active proton extrusion, is thought to produce a permissive state for proliferation [64]. In general, cytoplasmic pH (pH_i_) is a most potent modulator of cell function. Displacements of pH_i_ from resting levels (7.0–7.5) can arrest cell growth and induce apoptosis [63,64,65]. It has been proposed that tumor cells may have a dual benefit from increased proton extrusion. First, pH_i_ is maintained within proliferation-permissive values irrespective of intense glycolysis. Second, extracellular acidification facilitates the activity of the matrix proteases that promote cell migration and invasiveness [63]. Recent work from S. Reshkin’s research group contributed to highlight the fundamental role played by the tumor extracellular metabolic microenvironment during malignant invasion. Extracellular environment is mainly acidified by the Na^+^/H^+^ exchanger NHE1 and the H^+^/lactate cotransporter that are typically active in cancer cells. What is more, NHE1 also regulates formation of invadopodia—cell structures that mediate tumor cell migration and invasion. The NHE1 located at invadopodia acidifies the local extracellular nanoenvironment in order to drive protease-dependent and -independent proteolysis of the extracellular matrix (ECM) proteins, thus permitting invasion to occur. The invadopodial ECM digestion modulated by extracellular pH is also stimulated by serum deprivation, by hypoxia and EGF. The latter stimulates invadopodial digestion and particularly the NHE1-dependent acidification of the peri-invadopodial nanospace. These observations provide a starting place to figure out the mechanisms by which the tumor microenvironment and growth factors interact to drive tumor progression [27,63,65,66,67,68,69,70].

Not surprisingly, considering the above evidence, carbonic anhydrase (CA) has also attracted increasing work in cancer biology, particularly the membrane-bound CA forms [65]. Special attention has been devoted to CA-IX, a hypoxia-inducible, tumor-associated extracellular-facing CA implicated in pH control of colon, bladder and breast cancer [71,72,73]. It has been proposed that CA-IX activity helps to vent CO_2_ from respiring cells, by hydrating extracellular CO_2_ to HCO_3_^-^ and H^+^. Such a facilitated CO_2_ diffusion maintains a steep outward CO_2_ gradient, with alkaline pH_i_ and acidic pH_e_. Such an environment may contribute to stimulate tumor growth and invasiveness. Tumors that do not express CA-IX or other extracellular-facing isoforms (e.g. CA-XII) may resort to different transport mechanisms for proton extrusion, such as lactic acid efflux through monocarboxylic acid transporters (e.g. MCT4, a hypoxia-induced gene-product). The expression and hypoxia-inducibility of CA-IX of different cancer cell lines may provide information about their specific pH regulation strategies.

## 3. Possible Approaches for Ion Channel Targeting in Oncology

In principle, drugs can affect ion channels through several different mechanisms [74,75]. They can produce direct channel inhibition by blocking the pore or by obstructing the agonist binding site. Moreover, they can modify the channel residence in the different conformational states, by interacting with allosteric sites. Channel proteins are also affected by the composition and physical state of the plasma membrane, which can be also modified by several drugs. Finally, it is increasingly recognized that ion channels form complexes with a variety of membrane proteins, which suggests other possible ways for altering channel function. Many channel inhibitors exert their effects on the extracellular side, which usually makes the treatment is easier to calibrate and decreases aspecific metabolic effects.

Choosing an oncologic therapeutic strategy requires considering the balance between two sometimes conflicting issues. A drug must produce potent and specific inhibition of a given channel type, in order to damage the targeted cell type, without causing important toxic effects in other tissues expressing the same or related channels. Several ways to obtain this goal are possible. In the case of voltage-gated channels, different cell types may express different proportions of channels in the different conformational states, because of their different V_m_ dynamics. Therefore, tissue specificity can be obtained by using compounds that preferentially bind these states, even when they poorly discriminate between channel subtypes. Such an approach has been considered for treating neuropathic pain by selectively blocking the VGSCs expressed in depolarized cells, a high proportion of which resides in the inactive state [76]. This requires detailed understanding of the mechanism of drug action, which is however relatively easy to obtain with the modern methods of cell physiology, especially patch-clamp. The latter technique is also invaluable for testing the mechanism of action of newly synthesized compounds, whose design [77,78] should be considerably facilitated by the rapid advances in the determination of the three-dimensional structures of ion channels [79,80]. The details of drug action are more easily carried out on ion channels expressed in heterologous systems. The relevant results can be next confirmed in more physiological preparations, such as primary cultures or brain slices. In this way, detailed mechanistic insight as well as evaluation of the drug’s effects on cell processes such as action potential firing, muscle contraction and secretion can be obtained, which is necessary to assess the possible side effects. The possibility of combining biophysical and pathophysiological studies in intact cells makes ion channels very appealing for rational drug development and screening. Nonetheless, targeting ion channels is still an under-exploited therapeutic strategy. The recent development of high-throughput automated electrophysiological methods should give considerable impulse to the field. In fact, new compounds with channel-modulating activity have been recently used to treat a variety of disorders (e.g. [81]). However, the side effect issue remains a general problem. Below, we discuss a few exemplary cases regarding both ion channels and ATPase transporters.

**Table 1:** Some ion channels and transporters relevant in oncology.

Channel type	Function	References
**K^+^ channels:**	Cell proliferation	[1,2,3,28,29,30,31,32,34]
K_V_	Cell invasiveness	
	Chemoresistance	
	Angiogenesis	
	Chemical cancerogenesis	
K_Ca_	Cell proliferation	
	Cell volume	
	Cell migration	
K_IR_	Cell proliferation	
K_2p_	Cell proliferation	
**Ca^2+^ channels:**	Cell proliferation	[3,11,12,13]
Ca_V_	Cell proliferation	
SOC	Apoptosis	
**Na^+^ channels:**	Cell migration	[3,35,36,37,38,39]
Na_V_	Cell invasiveness	
**TRP channels**	Cell proliferation	[3,14,15,16,17,18,19]
	Apoptosis	
	Cell volume	
	Angiogenesis	
**Cl^-^ channels**	Cell volume	[3,20,21,22,23,24,25]
	Cell migration	
**nAChR**	Cell proliferation	[40,41,42,43,44,45,46,47]
	Apoptosis	
	Angiogenesis	
	Cell invasiveness	
**Aquaporins**	Cell cycle control	[48,49,50,51,52,53,54,55]
	Angiogenesis	
**Sodium pump**	Cell migration	[56,57,58,59,60,61]
	Cell invasiveness	
	Chemoresistance	
**Na^+^/H^+^ exchanger (NHE1)**	Tumor cell metabolism	[26,27,63,66,67,68]
	Cell invasiveness	
	Chemoresistance	
**Carbonic Anhydrases (CA-IX, CA-XII)**	Tumor cell metabolism	[65,71,72,73]
	Cell growth	
	Cell invasiveness	

The listed examples refer, with no pretension of exhaustiveness, to the proteins under active study in the field and mentioned in the main text. Table also includes the membrane-bound carbonic anhydrases, which are also mentioned in the text. In addition, the references include a few recent reviews expanding on themes only cursorily treated here (e.g. [3] and [63]).

## 4. The Case of K_v_11.1 (hERG1 or KCNH2): Specificity and Side Effects

K_v_11 (also known as ERG, from *Ether-à-go-go* Related Gene, hERG in humans) is a family of voltage-gated channels that comprises three main subtypes (K_v_11.1, K_v_11.2 and K_v_11.3). The broad physiological properties of these subtypes are similar, although the specific expression and role in different tissues is debated. Because of their voltage dependent properties, K_v_11 channels can exert different physiological functions. First, they contribute to shape the action potential repolarization, as is typically the case in heart myocytes. These channels activate/inactivate during the long depolarized plateau. Subsequently, during the repolarization phase, they quickly recover from inactivation, thus transiently opening before they close again (deactivate, more precisely) at negative V_m_. Second, the steady state properties of K_v_11 are such that a significant fraction of these channels is open at V_m_’s around −40 mV. These channels can thus modulate excitability and contribute to the resting V_m_ of non-excitable cells, although the precise kinetic features varies between K_v_11 subtypes [82]. In endocrine cells, these channels control the firing frequency, and thus hormone release, as has been shown in pituitary lactotroph [83], β-pancreatic [84] and chromaffin cells [85]. In general, K_v_11 is widely expressed in the mammalian central nervous system (CNS, [86,87]) and its role in the regulation of neuronal excitability has been demonstrated in a variety of adult and developing rodent preparations [88,89,90,91,92]. Moreover, recent work has revealed a primate-specific cerebral K_v_11.1 isoform (KCNH2-3.1) that controls neuronal excitability and seems to be implicated in neurological disorders [93]. 

However, current evidence indicates that the most serious side effects observed when administering hERG inhibitors to patients are not caused by endocrine or neurological alterations, but by cardiac arrhythmias. In humans, hERG channels are thought to be the molecular correlate of the pore-forming subunit of the cardiac I_Kr_ current, which contributes to the action potential repolarization phase, for the biophysical reasons illustrated above [94]. Blocking K_v_11.1 retards the cardiac repolarization, which is reflected in prolongation of the electrocardiographic QT interval. Uncontrolled QT interval lenghtening can result in *torsade de points* (TdP), a life-threatening ventricular arrhythmia that may lead to ventricular fibrillation [95]. In fact, *hERG* mutations can cause the long QT syndrome [96]. Many K_v_11.1 blockers, such as E4031, Way 123,398, dofetilide and others belong to the class III antiarrhythmic drugs, which can lead to fatal arrhythmias. During the last eighteen years, reports of QT prolongation (associated with hepatotoxicity) have in fact determined more than 60% of drug withdrawals [97]. In addition, the cardiac side effecs presented by many hERG1 blockers in humans are often accompanied by an unwieldy blocking mechanism, since they bind the intracellular channel face.

These features of K_v_11.1 inhibitors are unfortunate, because ample evidence indicates that hERG expression/activity is implicated in neoplastic progression. In fact, studies *in vitro* as well as *in vivo* suggest that hERG1 blockers are worth considering for oncological therapy. Blocking K_v_11.1 tends to arrest cell proliferation in a variety of cultured neoplastic cells [29,31,32,98,99,100], blocks the invasiveness of colorectal cancer cells [28] and the VEGF-A secretion from cultured glioma cells [101] and myeloid leukaemia cells [29]. What is more, very recent evidence indicates that similar effects are obtained *in vivo,* as treatment of immunodeficient mice with E4031 decreases (i) the growth of exogenous human gastric and colon cancer cells and (ii) the bone marrow engraftment and peripheral blood invasion of myeloid or lymphoblastic leukaemia cells [6,7]. In the following paragraphs, we discuss possible ways to circumvent the problems that therapeutic use of K_v_11.1 blockers may cause.

### 4.1. Not all K_v_11.1 blockers produce arrhythmias

Apart from class III anthyarrhythmics, K_v_11.1 is blocked by many other compounds, such as some antihistaminics (e.g., terfenadine), prokinetics (e.g., cisapride), antipsychotics (e.g., sertindole) and antibiotics (e.g., erythromycin). This molecular ‘promiscuity’ resides in the structural features of the intracellular channel cavity, to which most of these drugs bind [97,102,103,104]. What is interesting from our perspective is that not all of these compounds produce arrhythmogenicity [105]. Verapamil, for example, produces strong hERG inhibition without prolonging the QT interval in both animals and humans. Probably, the effect caused by hERG block is balanced by a concomitant reduction of action potential duration produced by the blockade produced by verapamil on voltage-dependent Ca^2+^ channels [106]. Other drugs, such as the antipsychotic sertindole, inhibit K_v_11.1 and prolong QT, without inducing torsadogenic effects [102]. The torsadogenic potential of channel blockers is generally unknown, because of the limited knowledge we have about the precise mechanistic relationship between QT prolongation and arrhythmia. Because sertindole has very high affinity for K_v_11.1 [107], it is possible that the higher torsadogenicity of other hERG blockers depends on their lower specificity, which may cause supplementary effects on other ion channels. Therefore, blocking hERG does not necessarily produce fatal arrhythmias and the structure of sertindole may suggest how to synthesize even more specific compounds. This example shows the necessity of obtaining a complete profile of the effects of the most promising drugs on different ion channels. This problem often arises when targeting ion channels. For example, antiepileptic drugs usually exert their action by modulating ion channels. Nevertheless, the mechanism of action is often poorly understood, because the full spectrum of molecular targets of these compounds is generally unknown [108]. To plan rational pharmacological strategies, mechanistic studies should be followed by further tests of the physiological effects on tumor as well as other cell types.

### 4.2. Compounds that bind different conformational states or different channel regions

The ion channel function is characterized by continuous transitions between a few relatively stable conformational states, such as open (active), closed (deactivated) and inactivated (or desensitized, in ligand-gated channels). Therefore, when different cell types have distinct V_m_ dynamics, the same channel isoform may present a very different distribution of conformational states. Because many drugs bind conformational states with different efficacy, they may selectively target certain cell types simply because they tend to bind different channel states. This strategy is currently considered in treatment of neuropathic pain [76]. Compounds such as lamotrigine and lidocaine preferentially target open and inactivated voltage-gated Na^+^ channels, without distinguishing the different subunits. The damaged neurons responsible for neuropathic pain are abnormally depolarized, so that the time spent by Na^+^ channels in the open or inactive state is much longer than it normally is in excitable cells. Therefore, cumulative channel inhibition is considerably more effective in damaged neurons.

**Figure 1:**
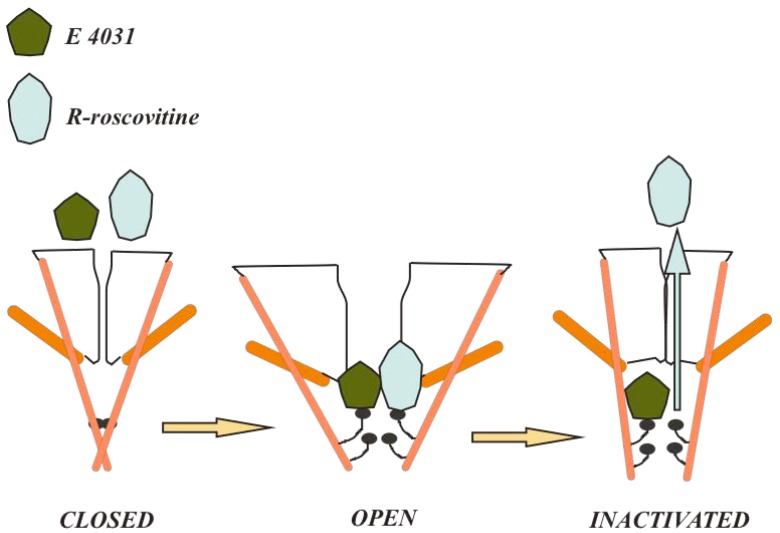
Differences in the state-dependent block of hERG1 channels by R-roscovitine and class III anthyarrhythmic drugs (E 4031).

Similar methods could be applied to cancer therapy. Neoplastic cells are often rather depolarized and their changes in V_m_ are usually slow, even when these changes oscillate in phase with the cell cycle stages. Therefore, the proportion of time spent by a voltage-gated channel in a given state can be very different in tumors and excitable cells. A recent example that points to the feasibility of this approach is offered *R*-roscovitine [109]. Roscovitine is a cyclin-dependent kinase inhibitor currently tested in phase II clinical trials as an anticancer agent. *R*-roscovitine quickly and reversibly blocks hERG at clinically relevant concentrations. Data suggest that this drugs blocks the open channel, which may explain why it does not produce arrhythmic effects (see Figure 1). HERG is usually open for only a transient phase during the cardiac action potential repolarization, whereas during most of the cardiac lifetime, K_v_11.1 is either deactivated (at negative V_m_) or inactivated (during the depolarized plateau). Thus, not enough time is available to open channel blockers, at appropriate concentrations, to produce significant hERG block in cardiac cells. On the other hand, a significant fraction of open K_v_11.1 channels should be present in proliferating cells, with typical resting V_m_ around −40 mV. Hence, inhibitors that preferentially bind the open channel might produce significant cumulative effects on K_v_11.1-expressing tumor cells, without causing significant side effects on cardiac myocytes. Roscovitine, in particular, also provides the possibility of simultaneous targeting cyclin-dependent kinases and hERG, with potentially cumulative effects. Recent studies have also addressed the frequency-dependence of hERG1 blockade for different compounds, which offers further insight on the possible effects on excitable cells [110]. The notions illustrated above can be extended to the other VGKCs for which drugs addressing specific conformational states are known [111,112].

### 4.3. Peptide toxins: accessibility from the extracellular side

A supplementary problem presented by many of the K_v_11-targeting inhibitors is that they often bind to the intracellular side of the channel protein. To develop more convenient drugs a possible starting point is the structure of the several peptide toxins recently found to bind different extracellular channel portions, with high specificity. For example, the scorpion toxins BeKm-1 and CnErg1 (ErgTx1) bind the external vestibule when the channel is closed. In this way, they shift the ERG activation curve to the right (more positive). When the channel is open, these peptides also obstruct the channel pore, to different extent, with BeKm-1 probably targeting a deeper pore region [113,114]. Contrary to the above peptides, the sea anemone toxin APETx1 is specific for K_v_11.1 [115]. It alters gating by interacting with the S3b helix, instead of the channel vestibule [116]. Further studies on these peptides could suggest how to develop state-specific drugs fit for therapeutic use. For review about ERG toxins and their mechanisms of action see Wanke and Restano-Cassulini [117]. Supplementary isoform-specific scorpion toxins have been discovered recently [118,119]. For example, five peptides were purified from the venom of *Grammostola rosea* tarantula. These toxins exhibit different degrees of specificity for tetrodotoxin-sensitive Na^+^ channels and the three main isoforms of K_v_11, but do not block *Shaker*-related K^+^ channels. Interestingly, these peptides produced both open channel blockade and gating modifications at different concentration ranges. Therefore, it is possible in principle to calibrate a blocker’s concentration to obtain the most useful effect.

## 5. Recent Oncological Applications of Ion Channel and Transporter Blockade

We recently reviewed the full spectrum of potential methods to obtain channel inhibition in a way applicable to cancer therapy [3]. In the following paragraphs, we briefly illustrate a few approaches that have already achieved some success (see also Table 2).

### 5.1. Specific targeting of ion channels with different methods

Because in some cases individual channel types can be assigned to specific cancer types [3], it is now possible to seek highly specific blockers to target specific tumor tissues. In effector/memory T (T_EM_) cells, K_v_1.3 function is implicated in inflammatory autoimmune diseases such as multiple sclerosis and rheumatoid arthritis [120,121]. Chandy and coworkers recently obtained highly specific targeting of K_v_1.3 with a modified sea anemone toxin (ShK-186). This treatment produces long-term loss of T_EM_ cells in mice, with ensuing improvement of autoimmune disease symptoms. Moreover, the toxin spares the T-cell population that confers protection against infection and cancers [122]. However, T_EM_ cells exert protective effects only in some cancer types, but not others [123,124,125]. Therefore, prolonged treatment with K_v_1.3 blockers must be carefully evaluated in each case.

A recent example of the potential usefulness of specific channel-targeting peptide toxins as anti-cancer compounds is offered by studies in lung cancers. α-Cobratoxin specifically inhibits α7 nAChRs, those more involved in uncontrolled proliferation. This toxin was found to exert antitumor effects after xenografting either pleural mesothelioma cells [126] or NSCLC cell lines [127] into immunodeficient mice.

In metastatic prostate cancer, promising results have been obtained by using novel blockers for VGNCs with high potency and minimal acute toxicity. These compounds have been used *in vitro* [128,129] and in human prostate cancer xenografts [130]. 

Specific ion channel targeting can also be obtained by developing monoclonal blocking antibodies. Although several such tools have been recently produced [131,132], this approach is still in its infancy, as far as cancer therapy is concerned. Use of a monoclonal antibody against K_v_10.1 (EAG-1) channels is described is the next paragraph.

Finally, specific inhibition can be obtained by using antisense oligonucleotides and small interfering RNAs. Concrete suggestion that this method could be useful in oncology comes from work on the intracellular Cl^−^channel CLIC4, whose expression increases in keratinocytes after exposure to DNA-damaging compounds. Expressing inducible antisense nucleotides against CLIC4 has been shown to stimulate apoptosis in a squamous cancer cell line. When these cells are transplanted into nude mice, when the CLIC4 level is reduced by expressing an inducible CLIC4 antisense oligonucleotide, cells undergo apoptosis. In tumors derived from transplanting these cells into nude mice, application of the antisense oligonucleotide inhibits tumor growth by increasing apoptosis and reducing proliferation, an effect potentiated by TNFα [133].

**Table 2:** Possible approaches to target ion channels and transporters in oncology.

Approach	Examples	References
Specific non-peptide inhibitors	K_v_11.1	[6,7]
	VGNCs	[128,129,130]
	Na^+^ /K^+^ ATPase	[134,135],
Targeting channel states	VGNCs	[76]
	K_v_11.1	[109]
Use of peptide toxins	K_v_11.1	[119]
	K_v_1.3	[122]
	nAChRs	[126,127]
Blocking antibodies	K_v_10.1	[136]
Antisense oligonucleotides / siRNAs	CLIC4	[133]
	nAChRs	[127]
		
Delivering cytotoxic compounds	K_v_10.1	[136]
	ClC-3	[137]

Table summarizes the main possible approaches for targeting ion channels and transporters, with possible oncological relevance. The listed examples specifically refer to some channel and transporter types under active study in the field and mentioned in the main text. The references include a few recent reviews expanding on themes only cursorily treated here (e.g. [119]).

### 5.2. Using channel-specific toxins and antibodies to deliver cytotoxic compounds

K_v_10.1 channels are significantly expressed outside the central nervous system only during the progression of particular tumors. No specific inhibitor is known against these channels. Therefore, monoclonal antibodies have been generated, which offer the advantage of being highly specific for K_v_10.1 and being unable to cross the blood-brain barrier. Although these antibodies display moderate anti-tumor efficacy, they can be very useful for diagnostic purposes and for local delivery of cytotoxic drugs [136]. A complementary approach is using monoclonal antibodies against tumor-specific membrane proteins to deliver ion channel blockers on a given neoplastic tissue. This method avoids widespread effects of the blocker on other tissues [138,139]. A successful example is targeting of the ClC-3 channels that are implicated in human glioma spread. These channels can be indirectly inhibited by chlorotoxin (Cltx), a scorpion toxin that binds a membrane-bound metalloproteinase. This latter can move within the plasma membrane and thus inhibition of ClC-3 is produced indirectly [137]. A synthetic Cltx derivative labelled with I^131^ has recently completed phase I clinical trials for patients with high-grade gliomas [23] and a multi-centre phase II trial is currently in progress.

### 5.3. Na^+^/K^+^ ATPase

Cardiotonic steroids (cardenolides) are typical natural inhibitors of the Na^+^/K^+^ ATPase. Their anti-cancer effects are usually too weak to allow them to be used clinically at reasonable doses. However, a recently developed hemisynthetic derivative of the cardenolide 2”-oxovoruscharin (UNBS1450) binds to the pump’s α1 subunit with 1 to 2 orders of magnitude higher affinity than that displayed by the classical cardenolides. Treatment with UNBS1450 leads to cell death in gliomas overexpressing the Na^+^ pump and, i*n vivo*, prolongs the survival of mice orthotopically grafted with glioblastoma cells [60,61]. This compound also produces a marked inhibition of NSCLC cell growth *in vitro* [57]*.* Its efficacy *in vivo* is presently under study in preclinical trials [56]. The probable mechanism of action on glioblastomas is inhibition of cell migration through disorganization of the actin cytoskeleton and stimulation of proautophagic effects. Very recent results indicate that UNBS1450 also exerts anti-tumor effects on melanoma cells both *in vitro* and *in vivo* [134] and seems also very effective on multi-drug-resistant cancer cells, generally refractory to chemotherapy [135].

## 6. Conclusions

Ion channels are still somewhat neglected as therapeutic targets, except in typical diseases of excitability processes, such as epilepsy and cardiac arrhythmias. This is somewhat unfortunate because ion channels may offer considerable advantages in terms of mechanistic understanding and clinical potential. A growing body of evidence is determining the specific channel expression and roles in many tumor types [1,2,3,140]. As a consequence, several clinical trials are already in progress for a few channel-targeting compounds. We believe the evidence reviewed here indicates that more widespread efforts should bring interesting pharmacological applications of ion channel (or transporter) inhibitors in oncology.
